# Propagation of Medicinal Plants for Sustainable Livelihoods, Economic Development, and Biodiversity Conservation in South Africa

**DOI:** 10.3390/plants12051174

**Published:** 2023-03-03

**Authors:** Olufunke O. Fajinmi, Olaoluwa O. Olarewaju, Johannes Van Staden

**Affiliations:** 1 Department of Nature Conservation, Faculty of Natural Sciences, Mangosuthu University of Technology, Durban 4031, South Africa; 2 Research Centre for Plant Growth and Development, School of Life Sciences, University of KwaZulu-Natal Pietermaritzburg, Private Bag X01, Scottsville 3209, South Africa

**Keywords:** South African medicinal plants, red data list, biodiversity conservation, plant propagation

## Abstract

South Africa is blessed with vast plant resources and unique vegetation types. Indigenous South African medicinal plants have been well-harnessed to generate income in rural communities. Many of these plants have been processed into natural products to heal a variety of diseases, making them valuable export commodities. South Africa has one of the most effective bio-conservation policies in Africa, which has protected the South African indigenous medicinal vegetation. However, there is a strong link between government policies for biodiversity conservation, the propagation of medicinal plants as a source of livelihood, and the development of propagation techniques by research scientists. Tertiary institutions nationwide have played a crucial role in the development of effective propagation protocols for valuable South African medicinal plants. The government-restricted harvest policies have also helped to nudge natural product companies and medicinal plant marketers to embrace the cultivated plants for their medicinal uses, and thus have helped support the South African economy and biodiversity conservation. Propagation methods used for the cultivation of the relevant medicinal plants vary according to plant family and vegetation type, among others. Plants from the Cape areas, such as the Karoo, are often resuscitated after bushfires, and propagation protocols mimicking these events have been established through seed propagation protocols with controlled temperatures and other conditions, to establish seedlings of such plants. Thus, this review highlights the role of the propagation of highly utilized and traded medicinal plants in the South African traditional medicinal system. Some valuable medicinal plants that sustain livelihoods and are highly sought-after as export raw materials are discussed. The effect of South African bio-conservation registration on the propagation of these plants and the roles of the communities and other stakeholders in the development of propagation protocols for highly utilized and endangered medicinal plants are also covered. The role of various propagation methods on the bioactive compounds’ composition of medicinal plants and issues of quality assurance are addressed. The available literature, media online news, newspapers, and other resources, such as published books and manuals, were scrutinized for information.

## 1. Introduction

South Africa is blessed with abundant natural resources, as nearly 10% of all flowering plant species known to humankind exist in South Africa [1]. This is a valuable resource, and around 24,000 plant species have yet to be fully explored for the benefit of humankind [1]. Despite rapid urban and infrastructure development, increased Westernization, and access to conventional western medical facilities, the use of traditional medicine to treat diseases remains high in South Africa [2]. The value of traditional medicinal products extends from the prevention or cure of health problems to their cultural use, for purging and cleansing the body of evil spirits [3] and various other cultural purposes, thus making medicinal plants an important part of South African culture.

The functionality of the whole African traditional medicine system is solely dependent on the availability and sustainability of these endemic plants, and thus the propagation of medicinal plants is a crucial factor to ensure that such plants can be easily sourced when needed for treatment. In several African countries, sourcing medicinal plants from the wild are the norm. However, their propagation will relieve the pressure on the wild populations and conserve highly utilized, vulnerable, scarce, and endangered species. The use of medicinal plants to treat diseases and ailments is an integral part of African culture and is still being practised. Furthermore, the failing healthcare system in Africa makes the use of medicinal plants for health purposes an alternative and well-accepted practice, irrespective of financial status, class, and race. Traditional medicine trade in South Africa is a widespread growing industry, with an estimated income of about R2.9 billion per year, representing about 5.6% of the health budget for the country, with around 27 million consumers [4]. Therefore, it is necessary to put measures in place to sustain medicinal plant production to ensure their availability whenever the need arises. This will ensure that valuable medicinal plants are not lost and that knowledge about their potential is retained across generations. Hence, this review aimed to examine the status quo of highly utilized and traded South African medicinal plants and their propagation techniques. The objectives of the study were to review:

1. Plants that are highly potent and utilized, endangered, declining, incorporated into natural products, have cultural relevance, and contribute to the sustainability of livelihoods;
2. Factors involved in the propagation of plants resulting from objective 1 and issues related to quality assurance of plant materials sourced from propagation under controlled environments.

## 2. Results and Discussions

**Figure 1:**
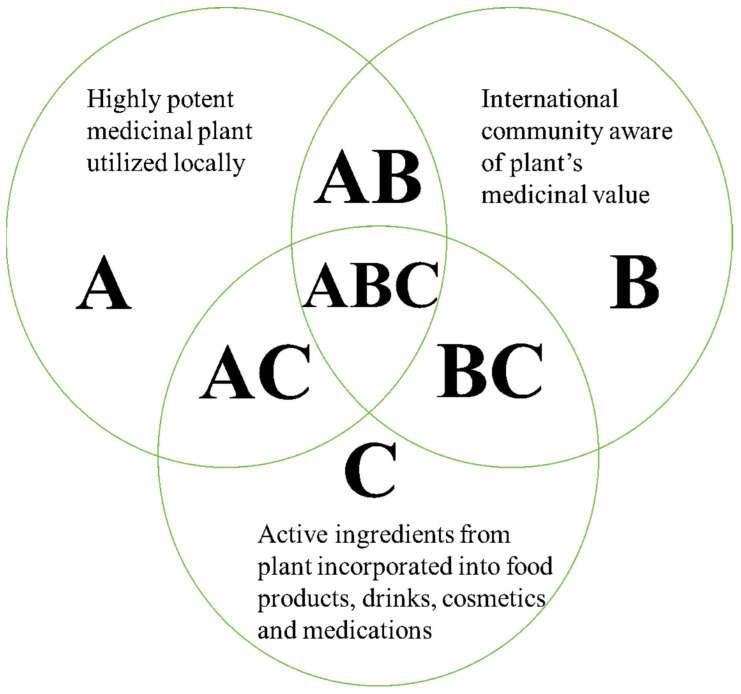
Factors responsible for the propagation of South African indigenous and naturalized medicinal plants.

**Figure 2:**
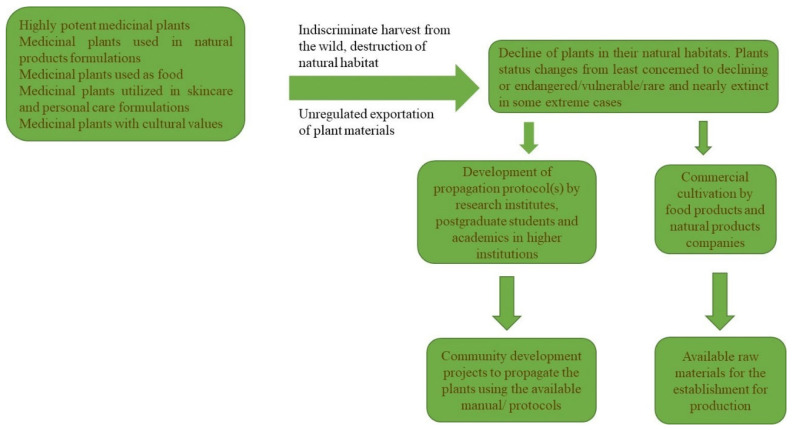
The path leading to the propagation of South African indigenous and naturalized medicinal plants.

The propagation of several South African indigenous plants has been the result of several factors, including government policies, community development projects, food purposes, exportation, and cultural practices. Other factors include in-depth research into the extraction of active ingredients of plants and the sale of medicinal plants to sustain livelihoods (Figure 1 and Figure 2), in parts or synergistic combinations. Several medicinal plants of South African origin are featured in the literature, because of their high potency for the cure of various diseases and infections. While some of these plants are restricted to South Africa, others are endemic to southern Africa, as their distribution cuts across southern African countries. This review features some of the most popular, highly utilized, and valuable plants with medicinal value within the southern African region. These include *Agathosma betulina* (Berg.) Pillans (buchu), *Aloe ferox* Mill. (bitter aloe), *Hoodia gordonii* (Masson) Sweet ex Decne. (hoodia, ghaap), *Hypoxis hemerocallidea* Fisch.Mey. and Avé-Lall. (“Africanpotato”), *Pelargonium sidoides* DC (“Umckaloabo”), *Siphonochilus aethiopicus* (Schweinf.) B.L.Burtt (African ginger), *Warburgia salutaris* (Bertol.f.) Chiov (pepperbark tree), *Sclerocarya birrea* (A. Rich.) Hochst. subsp. *caffra* (Sond.), *Cyclopia genistoides* (honeybush tea), and *Athrixia phylicoides* DC.

### 2.1. Medicinal Plants of Interest, Their History, Distribution, and Potential

#### 2.1.1. *Hoodia Godonni*

*Hoodia gordonni* is a cactus-like succulent of the Kalahari Desert (KD) in the southern part of Africa [5], and has an abundant distribution in southern Africa [6]. *Hoodia* is a genus of succulent plants widely used traditionally by the San people of southern Africa to quench thirst and suppress appetite [7], and has been consumed by the San hunters and gatherers for ages, especially during long hunting trips [8] and famine [5]. As a result of the knowledge of its indigenous use, a natural anti-obesity agent from *Hoodia* has been commercialized, leading to a growing demand from the international market for locally sourced *Hoodia* material [9]. Hence, the cultivation of the plant in South Africa and neighboring countries such as Namibia and Botswana, where *Hoodia* species also occur naturally, is a potential source for the generation of income for rural communities and households [9].

*Hoodia* is used as a traditional medicine to cure tuberculosis, indigestion, hypertension, severe abdominal cramps, hemorrhoids and diabetes [7], as well as a treatment for obesity [8]. The plant is attractive and a potential candidate for aesthetic purposes but difficult to cultivate, and is now a protected species in southern Africa [7]. This means that permits are required for certain activities [7]. In 2008, there were more than 20 international patent applications/registrations on *Hoodia gordonii*, with several *Hoodia*-containing commercial products available in the market [9]. In 2010, the plant was on the Red Data List as ‘Endangered’ and the government has since strictly controlled its export [10].

#### 2.1.2. *Hypoxis hemerocallidea*

*Hypoxis hemerocallidea* Fisch., C.A.Mey., & Avé-Lall. (Family: Hypoxidaceae), also known as African potato, iLabatheka, iNkomfe, moli, or starflower, has shown promise for use as a medicinal plant and natural product in southern Africa [9,11]. The plant is endemic to southern Africa and can be gathered in the wild. It has the potential to be developed into natural products and treatments due to its many medical applications [12]. Most varieties of the genus *Hypoxis* can be found in Africa, and tinctures, tonics, and lotions made from the species are among the products that can be bought on the market [12]. *H. hemerocallidea* is under tremendous stress because of being used as a “cure-all” treatment, and the population of the species is rapidly declining due to unsustainable harvesting, making it a strong candidate for conservation and propagation [12]. The only species of *Hypoxis* represented in the 51 plants of the African Herbal Pharmacopoeia is *H. hemerocallidea* [13]. Its corms are the most widely utilized part, making the plant susceptible to rapid destruction [14]. Because of this, conservation activities are now necessary due to the rapid decline in its natural population [13]. Hence, it was recommended that a platform be created to connect all stakeholders to manage *H. hemerocallidea* in the wild [12].

The popular therapeutic plant known as African ginger, *Siphonochilus aethiopicus* (Family: Zingiberaceae), is endemic to South Africa and is being overharvested for trade in the unofficial "muthi" marketplaces [15,16,17]. Due to commercial trade, the plant is now largely extinct, extremely rare, and designated as a critically endangered species in South Africa [17]. African ginger is used for a variety of purposes, which increases its demand. As a result, the plant may become scarce or even extinct in the wild, which is a severe issue for many stakeholders, especially traditional healers [17]. Fresh rhizomes are chewed to cure asthma, hysteria, colds, flu, coughs, pain, dysmenorrhea, influenza, hysteria, and malaria, and women chew them during menstruation [18]. Moreover, local companies use the plants to formulate tablets, capsules, and syrups for medicines [16]. The extremely scented roots have been believed to be utilized as a lightning repellent by the Zulu people [18]. Its cultural uses include spiritual healing, defence against evil spirits, and washing to fend off bad luck or evil spirits [17]. According to research conducted by [17], African ginger guards the divine bones (ditaola) from all evil spirits when a person arrives at the hut of a traditional healer with ulterior motives. Due to its geographic location and the presence of a wild population of the herb, Mpumalanga province of South Africa sees a high inflow of commercial plant gatherers from outside the community, which contributes significantly to overharvesting [17]. African ginger rhizomes are preferred to the root because they are easier to harvest and have better chances of surviving and regenerating [17]. However, cultivation of the plant is necessary for its long-term use. Mander, [2] estimated that, in KwaZulu-Natal, about 1.9 tons of African ginger, totalling 52,000 plants, are traded yearly. For many harvesters, the plant has provided a means of subsistence. Alternative strategies are continually being researched to ensure that the rising demand for African ginger is satisfied globally [19]. Wild ginger cultivation should be a feasible business in South Africa since there is always a need for it, and the cash earned compensates for the effort [20]. This is because street vendors earn roughly R140 per kg, while stores and healers get up to R450 per kg [2]. Large-scale cultivation is often the only option for many species to maintain the wild species as alive and economically viable [21]. Established micropropagation is not yet utilized on a significant scale for the proliferation of wild ginger [22,23]. 

#### 2.1.3. *Pelargonium sidoides*

*Pelargonium sidoides* DC. is another medicinal plant that is native to South Africa and mostly found along the coastline regions of the country, and in Lesotho [24]. A substance prepared from the root of the plant was used to cure tuberculosis in Europe during the first part of the 20th century [24]. One of the most popular phytomedicines in the world since the 1990s is EPs 7630 (Umckaloabo), an ethanolic extract of *P. sidoides* tuberous roots that is high in prodelphinidin. The extract was approved to treat respiratory tract infections, such as acute bronchitis [25,26]. The root is being harvested indiscriminately in very large quantities to meet the existing global demand, which is now unsustainable due to increasing harvests by many plant gatherers. This has now led to a downward trend in the demography of the species in their native habits [27]. Though there are no official statistics on annual harvests, it is believed that they range from 9000 to 45,000 kg per annum [24]. Germany alone generates more than € 80 million in sales annually [26]. A *P. sidoides* mother tincture mixture, known as Umkalor, is offered for sale in Latvia, Russia, and Ukraine [24]. Seven patents around the world are now covering the use of proprietary extracts of *P. sidoides* and associated preparations [24]. In South Africa, most of the plant material used to produce medicines is still collected by people from rural communities [28]. In traditional medicine, *P. sidoides* is used to cure a variety of illnesses, including those that cause a cough, fever, tuberculosis, sore throats, exhaustion, and general body weakness [27].

Although the South African Department of Agriculture has implemented the use of licenses to manage the wild harvests of *P. sidoides*, the practice is ineffective in stopping the unlawful gathering of the plant, especially from vulnerable areas [27]. The root is often damaged after harvest, compromising the possibility of vegetative regeneration. The plant naturally regenerates from seeds and perennates that grow through the underground root–tuber system [27] Therefore, it is recommended that vegetative multiplication be encouraged to preserve the plant in situ in the Eastern Cape, as well as in other natural habitats, and make available an alternative source of supplies [27].

#### 2.1.4. *Warburgia salutaris*

Several medicinal usages of *Warburgia salutaris* have caused it to become scarce due to its uncontrolled harvesting in the wild. This has led to about a 50% reduction in the South African population of the plant, with some subpopulations becoming almost extinct [29]. Several health issues can be treated with *W*. *salutaris*, but its bark is the most common part used, which is harvested by stripping it vertically from the tree. Senkoro et al. [30] proposed many conservation strategies to support the species’ sustainable usage, including the adoption of alternate species for the same use, the replacement of bark for leaves, and the cultivation of other species. Alternative plant material sources were proposed throughout the region as a main technique for species control [31,32,33]. Furthermore, the peppery scent of *W. salutaris* leaves makes them an appealing condiment for a variety of cuisine dishes and drinks [34]. Pepper bark is considered to be severely endangered in southern African countries due to frequent local subsistence uses and high commercial demand in urban centers [30,31].

#### 2.1.5. *Aloe*

*Aloe* is used for treating various diseases, such as gout, colon cancer, skin cancer, and thrombophlebitis, to stimulate immunity, and to treat diabetes, rheumatism, lung cancer, leukaemia, digestive candidiasis, and obesity [18]. *Aloe ferox* is also known as Cape Aloe, Bitter aloe, and Tap aloe, in Africa [35]. Red *Aloe* and Lily of the Desert is a species of *Aloe* indigenous to the south-eastern and western regions of South Africa’s Western Cape, Eastern Cape, and Free state [36]. *Aloe ferox* originated in South Africa and was also widely distributed throughout the tropics and subtropics, where it is grown as an ornamental and medicinal plant [35]. The popular *Aloe* gel is used to prepare aloe bitter powder, aloe drinks, aloe bitter crystals (for constipation), cosmetics, hair, and skin-care products [35].

#### 2.1.6. *Agathosma* spp.

*Agathosma crenulata* and *A. betulina* (also known as Buchu) are significant sources of essential oils, which are mostly extracted and utilized in the production of cosmetics, soaps, and food colorants, as well as pharmaceutically for the treatment of renal problems and chest complaints [37]. The two main oil constituents in the essential oils of *A. betulina* are iso-menthone and diosphenol, which are mainly sought after because of their antibacterial and diuretic properties [37]. It is generally known that the Khoisan people utilized essential oil for nearly everything, including treating their skin and stomach aches [37]. Buchu is now recognized as a protected species, as it is extremely vulnerable, to the point of extinction, and it is often referred to as the “abalone of the land” because of its commercialization. Since its primary applications are in the food and pharmaceutical industries, there is a significant demand for it on a global scale [38]. The Western Cape Nature Conservation Board (WCNCB) estimates that the buchu industry generates about R150 million annually [37]. Wild populations in the Western Cape mountains were the only sources of buchu before 1995, putting the resource at risk of extinction [37].

The Khoisan and other indigenous peoples referred to a variety of fragrant plants as buchu, and employed them in dance ceremonies, as anointing oil, cosmetics, perfume, and medicine [39,40]. Traditional knowledge belongs to the Khoisan within the scope of those documented usages and should be acknowledged and valued as such [40]. Buchu was a cherished Khoisan traditional medicine from an ethnobiological perspective, and it is now one of the most widely used herbal remedies in South Africa [41]. The traditional use of buchu includes the management of fever, rheumatism, gout, stomach disorders, kidney and urinary tract infections, and colds [41]. It was used externally as an antiseptic wash on infected wounds and as a compress for bruises, swelling, and sprains [41]. *A. betulina* is traditionally consumed orally as an aqueous infusion, occasionally sweetened with brown sugar, or as a tincture in brandy [41]. *Agathosma* species and other valuable fynbos species are on the conservation list [37].

#### 2.1.7. Cape Fynbos Endemic Tea, *Cyclopia genistoides*

The South African fynbos vegetation is renowned for its abundance and diversity of indigenous plant species that are adapted to acidic, nutrient-poor soils, formed from sandstone and shale, in a climate with cold, wet winters and scorching, dry summers [42]. Most fynbos plants are primarily harvested from the wild, whether they are utilized to produce natural herbal beverages, cut flowers, essential oils, or medications [43,44]. It is now obvious that sustainable cultivation is required because of the increased demand for these specialty commodities, which put a heavy strain on the natural areas where these resources occur [44,45]. 

There are endemic species of honeybush (*Cyclopia*) in South Africa’s Fynbos Biome of the Cape Floral Kingdom [46]. Nevertheless, its strength, in contrast to *Aspalathus linearis*, as a commercial crop lies in the variety of species that can be grown, its varied natural distribution, and the possibilities for wider habitat adaptation [47]. *Cyclopia genistoides* and *C. intermedia* are the most gathered species from the wild [46]. In the past two decades, honeybush and rooibos have gained popularity among health-conscious consumers. The herb, which does not contain caffeine, is used to make honeybush tea, a herbal infusion with a flavor reminiscent of honey [47,48]. To prepare herbal tea with a high concentration of antioxidants, honeybush leaf is traditionally chopped, fermented, and sun-dried [46]. In addition to its delightful flavor and aroma, honeybush tea has several health advantages, including antioxidant, anti-mutagenic, anti-cancer, and phytoestrogen qualities [49]. However, the International Union for Conservation of Nature’s Red List of Threatened Species lists the plant, because around 80% of them are harvested from the wild in an unsustainable manner [46]. Therefore, cultivation is necessary to ensure that enough tea is produced. However, the poor rooting responses of the species that regenerate from shoots represent a significant barrier to cultivation. The distinctive qualities of honeybush that contribute to its flavor, medicinal value, and processing have received most of the attention in research. However, much less is understood about plant breeding, pest and disease control, nutrient needs, harvesting techniques, and propagation [46]. 

#### 2.1.8. *Athrixia phylicoides*, the Bushman’s Tea

*Athrixia phylicoides* (Asteraceae) is indigenous to the northeastern mountains of South Africa, where it has been utilized to make tea, brooms, and traditional medicine [50]. The consumption of a refreshing beverage derived from dried leaves and twigs of *Athrixia phylicoides* is common in South Africa [51] and its popularity has led to over-exploitation of the plant [50]. The plant has a long history of use among the indigenous citizens of southern Africa, making it a possible candidate for commercialization [51], which could open new paths for the development of various food products. The bush tea plant has great potential in the cosmetic, food, and beverage industries [52]. Bush tea is used extensively as traditional medicine for the treatment of boils, sores, acne, cuts, infected wounds, headaches, colds, and loss of voice, as well as being used as a gargle for throat infections [53]. It has great potential as a garden specimen with its attractive mauve flowers [54]. Globally, South African tea is very popular and well sought after because of its valuable nutrients and medicinal values, making it beneficial for consumption. Similar to South African teas and tea products, Marula and its derivative products receive international interest due to its many functions.

#### 2.1.9. *Sclerocarya birrea,* the African Horticultural Tree with Immense Potential

*Sclerocarya birrea* (A. Rich.) Hochst. subsp. *caffra* (Sond.) Kokwaro, popularly referred to as Marula, is an African wild tree distributed across various African countries, where its parts such as leaves, stem bark, root, and fruits are utilized as food and traditional medicine [55,56] and are thus referred to as the “tree of life”. The fruit is high in vitamins C and K, as well as Na, Ca, Mg, Fe, Zn, and Mn, and sesquiterpene hydrocarbons, which are recognized for its antibacterial effects [57] The seed kernel, which is often consumed fresh or roasted [58], contains oleic, palmitic, myristic, and stearic acids, amino acids, glutamic acid, and arginine [55]. It is rich in oil and protein [55], with potential commercial oil production [58]. Marula is a crucial multifunctional plant in Africa [59], with its luscious fruit being an important component of southern African cuisines for centuries [60]. Because of its delicious fruit and medicinal properties, Marula has received great attention as a candidate for domestication and commercialization in southern Africa [61]. It has become a well-sought-after commercial crop, as the fruit pulp is used to make juice, alcoholic beverages [62], jams, and jellies [62,63]. Among all the products from Marula, only Amarula Cream (a popular liquor) has realized its full export potential, while an extract incorporated into cosmetic formulation has been patented by Phytotrade, in conjunction with Aldivia, a French company [24].

The Zulu people of South Africa use stem–bark decoctions of Marula to treat diabetes [64], and as enemas for diarrhea [65] and dysentery [66]. Traditional practitioners use decoctions as a wash before treating patients infected with gangrenous rectitis, which is also treated with the same decoction [67]. In different parts of Africa, chewing fresh leaves of Marula and swallowing the astringent juice is used for indigestion [68], while the stem bark is used to treat proctitis [69]. The plant also occurs across northern and western parts of Africa, with only the subspecies *caffra* existing in southern Africa [18]. Marula trees on communal lands are protected by the local chiefs because the plant is regarded as sacred [24]. The bark is utilized across Africa to treat various ailments/diseases, such as rheumatism, insect bites [24,66], malaria, and proctitis [70], while the aroma from the leaves relieves abscesses, burns, and spider bites. The oil is used to treat ear, nose, and throat conditions [24].

### 2.2. Effect of the South African Plant Red List Status on Medicinal Plant Propagation

According to the South African National Biodiversity Institute (SANBI), the critically acclaimed International Union for Conservation of Nature (IUCN) Red List is used as a criterion to prepare the South African plants’ Red List, which assesses the risk of extinction of species to emphasize the urgent conservation/protection requirement for these species. The IUCN system does not focus on species with a low risk of extinction but may, however, place a high importance on conservation. Hence, the Red List of South African plants is highly regarded as a basis for the implementation of South African conservation practices [7]. In addition, the Red List of South African plants is a crucial prompt for the propagation of medicinal plants, as it reflects the population decline, information on the oveharvesting of plant parts and the government ban on the harvesting of South African indigenous plants and other naturalized plants within South Africa. Over the years, the status of potent medicinal plants on the South African Red Data list frequently changes. For example, a highly utilized medicinal plant could have a status of ‘Least Concerned’ because of its wide range of distribution, and subsequent overharvesting from the wild could then drastically reduce its population, thus impacting the plant’s status. African ginger’s, *Siphonochilus aethiopicus,* status changed from ‘Not Threatened’ [71] to ‘Critically Endangered’ [72] and *Cyclopia genistoides’s* status changed from ‘Least Concerned’ [73] to ‘Near Threatened’ [74] (Table 1). Other factors, such as the exportation of raw materials and restricted distribution, pose a threat to some other medicinal plants. This is common with medicinal plants with restricted distribution to the Cape area only, such as the Karoo and other ecologically rich areas within South Africa. The government often places a harvest ban on plants with drastically declining populations. In addition, a plant with multiple uses is often a potential candidate in government conservation policies, which prompts the promotion of its propagation, in order to provide a protocol that could be used to propagate the plant to ensure its sustainability. For example, *Sclerocarya birrea* remains one of the most multipurpose traditional horticultural crops, with high cultural and medicinal relevance and valuable economic potential because of both its local consumption and international demands. The drinks produced from the tree are very popular and highly sought after globally. These potentials place *Sclerocarya birrea* in the ABC category illustrated in Figure 1.

**Table 1:** Red data list the status of some South African indigenous and naturalized plants, reasons for their propagation, and propagation protocols available in the literature.

Medicinal Plant	Red Data List Status and Reason for Propagation	Propagation Protocol
*Agathosma betulina* (P.J.Bergius) Pillans	Least Concerned The plant is highly harvested for essential oil with increasing demands and prices, resulting from demand from overseas markets. In 2006, legislation has been established to support the cultivation of plant materials and pressure on wild populations has declined [37].	Micropropagation and secondary metabolites in *Agathosma betulina* (Berg.) [75]. The biosynthesis of certain compounds increased in vitro, and in vitro seed germination yielded healthy seedlings [75].
*Aloe ferox*	Least Concerned. It is a medicinal plant of high commercial importance, as the leaves are extensively harvested, and a high quantity of materials has been exported since the 1980s, increasing in trade over the past 15 years [76]. Overexploitation in certain localities has led to localized extinctions [76].	[77] investigated the effects of temperatures, growth-promoting substances, and watering frequencies on seed germination and seedling growth of *A. ferox*. Smoke–water enhanced seed germination The cytokinins meta-Topolin (mT) and meta-Topolin riboside (mTR) at 5 mM gave significantly higher shoot multiplication rates compared with the control and benzyl adenine (BA)-treated plants, which gave a higher abnormality index.
*Athrixia phylicoides* DC.	Least Concerned	Investigation of the propagation and cosmeceutical application of *Athrixia phylicoides* [78]. Seed germination and vegetative propagation of bush tea (*Athrixia phylicoides*) protocol [79]
*Cyclopia genistoides* (L.) R.Br.	Least Concerned [73] and Near Threatened [74]. Populations are declining from overharvesting for tea production, as poaching of leaves and branches for tea occurs in several areas [74]. The lowland subpopulations in the Malmesbury, Cape Peninsula, Kleinmond, Hermanus and Albertinia regions have been lost to urban growth and in some cases, crop cultivation [74].	Woody rootstock regenerates after fire [74]. Honeybush can be propagated through both seeds and vegetatively [80,81], while the seed is the preferred method, as cuttings are expensive and difficult to root [82]. However, the seed germination rate in the genera could be low (≤15%) if not pretreated [80,81] with chemical scarification [81].
Hoodia	Not Threatened [71]. The species has undergone a drastic decline since 2001 from harvests, for its appetite-suppressant properties. International and national demands were especially huge between 2004 and 2006. There’s been strict enforcement/legislation to prevent wild harvesting in South Africa [83].	Unilever had patents for methods for the micropropagation of *Hoodia* plants in 2008 [84]
*Hypoxis hemerocallidea* Fisch., C.A.Mey. & Avé-Lall.	The *Hypoxis* population is declining in some subpopulations, especially in Gauteng, South Africa, because of extensive commercial exploitation, in addition to habitat loss and degradation [85]. Hypoxis is heavily traded at high prices [86]. Since it became popular in 1997 when an article in DRUM magazine referred to it as South Africa’s “miracle muthi”, being effective in strengthening the immunity of HIV sufferers [87]. The propagation of the plant is erratic because of seed dormancy and worsened by the fact that it does not propagate easily from corms [88].	[89] developed an ex vitro vegetative propagation technique for *Hypoxis hemerocallidea* corms. The author confirmed that the propagation method from the corms of *H. hemerocallidea* successfully produced cormlets and suggested that the upper corm parts could be used for propagation, while the bottom corm parts should be used for medicinal purposes [89] to achieve sustainable medicinal use of the plant.
*Pelargonium sidoides* DC.	Least Concerned. Plant remnants from harvests resprout well but the growth of the lignotuber is very slow, thus limiting subsequent harvests, in addition to declining populations [90]. The destructive harvests of the roots, which is the major plant part utilized for medicinal purposes, are the major cause of localized population decline [91].	A propagation protocol for *Pelargonium sidoides* from root cuttings has been developed [91], and a clonal propagation protocol for the plant was developed [27].
*Siphonochilus aethiopicus* (Schweinf.) B.L.Burtt	Critically Endangered [72]. The harvesting is unsustainable as the species population is rapidly declining, with extinction looming. As of 2000, 60% of the known extant subpopulations have less than 100 individuals [92].	A hydroponic propagation protocol for *Siphonochilus aethiopicus* [93] was developed. Efficient in vitro micropropagation and acclimatization protocols were developed to aid the conservation of the species’ [94] wild populations.
*Sclerocarya birrea* (A.Rich.) Hochst. subsp. caffra (Sond.) Kokwaro LC	Least Concerned. According to Moyo (2009), the increasing demands for Marula products by the food, pharmaceutical and cosmetic industries locally and internationally cannot be sustained by the wild populations.	[95] investigated the micropropagation and secondary metabolites of *Sclerocarya birrea.* The author observed a significant pharmacological activity in *S. birrea* renewable plant parts (leaves and young stems) and suggested plant part substitution as being a practical conservation strategy for this species.
*Warburgia salutaris (G.Bertol.) Chiov.*	Endangered [96]. There was at least a 50% decline in the South African population from the over-harvesting of bark for traditional medicine, particularly in KwaZulu-Natal [96].	Propagation protocol was developed for in vitro and ex vitro propagation [97].

### 2.3. Trends Leading to Medicinal Plants as Candidates for Propagation

Several medicinal plants’ statuses have evolved since their utilization and the exposure of European settlers and tourists to their potency and use locally (A). The knowledge of the potency transferred by European tourists to the international community (B) and their increasing popularity has led to the extraction of bioactive ingredients and their incorporation into food products, drinks, and others (C), making them potential candidates for bio-conservation and propagation (ABC; Figure 1).

The South African government’s policy of protecting indigenous flora has been very effective in the conservation of medicinal indigenous plants. South Africa has one of the best conservation policies in Africa. These policies have helped to curtail the overharvesting of medicinal plants that are in high demand. Over several decades, this has helped to protect these plants from the transition from their utilization by visiting European tourists to a stage where they are now more popular and in high demand. The strategical exploitation of South African endemic medicinal plants should involve the cultivation of the candidate plants to ensure sustainability (Figure 2). The sustainability of endangered and vulnerable species is often achieved through mass propagation. Micropropagation through plant tissue culture is a crucial tool for effective mass propagation. This technique has been used to develop an effective protocol for *Agathosma betulina* [75], *Aloe ferox* [77] *Hypoxis hemerocallidea* [98], *Pelargonium sidoides* [99], *Sclerocarya birrea* [100], *Siphonochilus aethiopicus* [101], and *Warburgia salutaris* [102] by the academic stakeholders. Community development projects to implement the mass production of valuable medicinal plants often use available propagation protocols developed by academics (Figure 2). However, the propagation of the plants is often not impactful, as the plants produced are not maximally utilized. Before the implementation of such projects, viable markets need to be identified, and products that can be utilized locally should be formulated and promoted by the Department of Science and Technology, while similar imported products should be placed on an import ban/restriction. Similar local products utilizing synthetic materials in their product formulations should be encouraged, in order to replace the synthetic products with natural ingredients from the plants. For example, Marula fruit is rich in vitamin C content, with a greater abundance than in oranges, and has abundant citric acid content. The latter is often used as a preservative in food product formulations. Hence, in-depth research into the potential use of Marula fruit as a preservative is advisable. 

### 2.4. Effectiveness of Propagation Method(s) of Medicinal Plants and Quality Assurance

**Figure 3:**
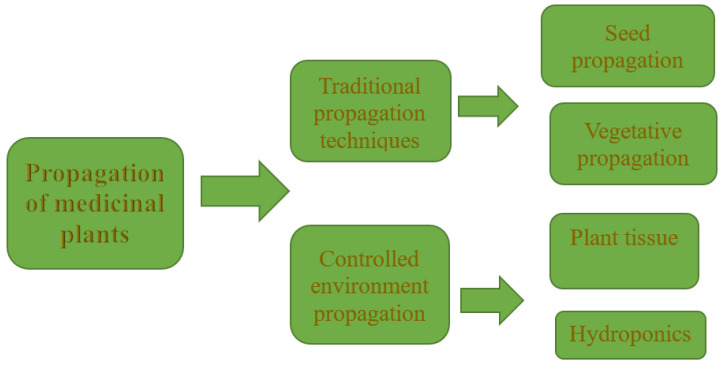
Propagation of medicinal plants in South Africa.

Various propagation methods have been used for the propagation of medicinal plants in South Africa (Figure 3). Medicinal plant nurseries, household gardeners and small-scale farmers often use the traditional propagation methods, which comprise seed propagation and vegetative propagation (Figure 3). However, these methods are often hampered by factors such as loss of seed viability, seed dormancy, and rooting difficulty of stem cuttings. The seeds of *S. birrea* [95] and *H. hemerocallidea* [88] have been reported to exhibit dormancy. Methods involving cold stratification, heat treatment, and the use of hormones to enhance radicle emergence have been adopted in seed germination protocols. Cold stratification enhanced the germination of *S. birrea* [95] and *Cyclopia* spp seeds [81]. These methods are used to mimic the natural environment of the plants in relation to the weather conditions that support seed germination on the field. The Fynbos vegetation in South Africa is species-rich, with various valuable medicinal plants endemic to the geological zone. This vegetation often experiences periodic fires, which often lead to the sprouting of seedlings from the soil seed bank. Hence, the use of smoke–water and heat, and the combinations of both, have been used in the seed propagation of various Fynbos species, such as *Agathosma* species [103] and *Aloe ferox* [77], among others. The use of smoke–water and its isolated compounds have now gained much popularity and are much utilized for the seed propagation of several medicinal plants. The use of smoke–water has been adopted by some small-scale farmers, as the information describing the successful germination of various South African medicinal plants is available on websites. 

According to Nwafor et al. (2021), various agronomic and agroecological challenges are a great hindrance to the successful propagation of medicinal plants by smallholder farmers [104]. These challenges include planting materials, extension support, markets, information, diseases, pests, support, agronomic, costs, and returns [104]. Progress in the propagation of medicinal plants by South African smallholder farmers and their involvement in its value chain can only be achieved when the complex socio-economic and socio-cultural challenges are well addressed. Refs [104,105] stated that the unavailability of seeds or propagation materials is among the challenges faced by small-scale farmers involved in medicinal plant propagation. The authors further stated that the cultivation of medicinal plants could contribute immensely to the financial empowerment of women in rural communities [105]. However, the propagation of plants through seeds and vegetative parts yields insufficient plant material for use as traditional medicine, especially in cases of endangered, vulnerable, or rare medicinal plants.

The functionality of the African traditional medicine system is solely dependent on the availability of adequate, quality plant material. Hence, the optimization of propagation methods of medicinal plants to ensure rapid, mass production of plant materials in vitro is a crucial tool that could fulfil high demands for valuable medicinal plants and is well embraced by plant conservationists in South Africa. Optimized propagation protocols developed by the academic stakeholders (tertiary institutions and research institutes) offer the opportunity to solve the problems associated with traditional propagation methods as previously highlighted. However, a high percentage of traditional medicine practitioners and users have, in the past, condemned the use of medicinal plants raised in vitro, because of the assumption that wild plants are natural and have better potency. 

Plant biotechnology is a vital and effective instrument that provides plant production options beyond those yielding high amounts of mass with quick production and can raise the levels of key bioactive compounds that are responsible for pharmacological activities (Table 2). However, some in vitro treatments have been reported to yield a decrease in some bioactive compounds. A Polish research team investigated the in vitro accumulation of valuable phenolic compounds of *C. intermedia, C. subternata* and *C. genistoides* [106,107,108]. The phenolic content of the shoots produced in vitro was similar to those produced in the shoots from the field. However, callus cultures did not produce mangiferin and isomangiferin, which are two of the major bioactive contents of the species. Factors, such as the effect of medium supplementation, temperature, and light regime on isoflavone production from *C. subternata* callus cultures, were investigated [106,107]. Medium supplementation with coconut water yielded increased biomass and high isoflavone production, while casein hydrolysate and phenylalanine supplementation had no favorable effect on either biomass or isoflavone formation. Higher temperatures and cold stress gave an increased isoflavone production but lower biomass during the first stage of growth, while the second stage of growth under the same conditions yielded an improved isoflavone production without any effect on the biomass. Culturing in the darkness, with coconut water supplementation, under dark conditions, yielded a significant increase in isoflavone production. The methoxylated isoflavones produced in *C. genistoides* shoot cultures were at low levels [108].

In the in vitro propagation of *Agathosma betulina*, the callus of nodal explants on medium containing 0.5 mg·L^−1^ NAA yielded the highest relative concentration of limonene with the absence of pulegone, making the treatment favorable for the in vitro production of limonene [103]. The production of hydroxybenzoic and hydroxycinnamic acid derivatives in *H. hemerocallidea* organ cultures was significantly increased by cytokinins, particularly the meta-topolin-treated organ cultures, which produced higher levels of gallic, protocatechuic, gentisic, and p-hydroxybenzoic, m-hydroxybenzoic, salicylic, chlorogenic and trans-cinnamic acids, while the isoprenoid cytokinins (N6-(2-isopentenyl)-adenine) significantly increased the production of hydroxycinnamic acid derivatives, namely, caffeic, p-coumaric, sinapic and ferulic acids [109]. The cytokinin-treated organ cultures displayed a significant increase in antioxidant activity [109]. However, cytokinin use in callus cultures decreased the concentrations of hydroxycinnamic acid derivatives and antioxidant activity when compared to the control [109].

This raises the issue of the suitability of biotechnology propagation techniques, as regards quality assurance. The use of hydroponics for the propagation of medicinal plants is not a common practice in South Africa, but researchers are gradually accepting the idea because of its positive outcomes. For instance, Giurgiu et al. 2014 [110] highlighted the improved concentration of bioactive properties in medicinal plants that are cultivated through hydroponics. Xego et al. 2016 [111] also observed that various hydroponic cultivation methods produced more shoots than traditional soil cultivation, coupled with comparatively higher yields of bioactive contents and total activity than the wild-sourced plants. Furthermore, [112] reported that plants grow faster in hydroponic conditions because they obtain all the nutrients they need in the proper amounts and proportions. The increase in watering intervals from 3 to 5 days during *S. aethiopicus* hydroponics propagation enhanced the levels of phenolic compounds [93]. Water deficit is known to have a positive impact on plant tissue secondary metabolite concentrations [113]. Hence, the successful propagation of medicinal plants is highly dependent on various environmental (natural or mimicked in vitro) biotic and abiotic factors, which can act singly or synergistically to modify essential oil and secondary metabolite contents.

**Table 2:** Major bioactive compounds of chosen medicinal plants and their pharmacological activities.

SN	Medicinal Plant Species	Major Bioactive Compound(s)	Pharmacological Activities and/or Mode of Action of Major Bioactive Compound(s)
1.	*Agathosma betulina* (P.J.Bergius) Pillans	*A. betulina* essential oil’s major components are (ϕ)-diosphenol, and (iso)menthone [114].	Menthone has sedating and antipyretic activities [115]
2.	*Aloe ferox*	Aloe-emodin, chrysophanol, and aloin A [36]	Aloe-emodin is an anticancer agent with selective activity against neuroectodermal tumours [116]. Aloe-emodin could kill GSDME-expressed cancer cells through pyroptotic cell death [117]. Aloe-emodin inhibited the growth of *Bacillus cereus, Bacillus subtilis, Staphylococcus aureus, Staphylococcus epidermidis, Escherichia coli, and Shigella sonnei* [36]. Chrysophanol has antidiabetic, anticancer [118] and hepatoprotective activities [119]. Kim et al. (2010) [120] reported that Chrysophanol inhibited the production of TNF-α and IL-6, as well as the expression of COX-2 upon treatment with LPS. Chrysophanol inhibits *B. subtilis, S. epidermidis, and E. coli* while aloin A inhibits all the tested bacterial strains [36].
3.	*Athrixia phylicoides* DC.	Bush tea is rich in flavonoids and tannins, and its major antioxidant compound, (6-hydroxy luteolin-7-O-β-glucoside) was isolated [121]. Monoterpenes and sesquiterpenes, such as α-pinene, β-pinene, caryophyllene oxide, β-caryophyllene, myrcene and spathulenol, were major components of the essential oil [122]	Luteolin and its glycoside have been reported to regulate NF-κB, MAPK, and JAK/STAT pathways, and to modify the effects induced by pro-inflammatory cytokines such as TLRs, TNF, IL-1TNF, IL-1, and IL-6 [123]. Luteolin controls glucose metabolism, cell growth, and the apoptosis process, which are often not regulated in malignant cells. Hence, luteolin is an anti-cancer compound [124] with potential treatment for diabetes [125]
4.	*Cyclopia genistoides* (L.) R.Br.	Xanthones and benzophenones [126]. Mangiferin.	Xanthone exhibits anti-inflammatory and anti-diabetic activities [127]. The occurrence of mangiferin with benzophenone α-glucosidase inhibitors in *Cyclopia* [128], and any other components that improve glucose uptake in vitro, such as iso mangiferin and scolymoside [129], makes *Cyclopia* extract a potential anti-diabetic nutraceutical [130].
5.	Hoodia	P57AS3 (an oxy pregnane glycoside) was isolated from *H. gordonii* and patented by CSIR South Africa [131].	The mechanism of action of P57AS3 for appetite suppression is by increasing the ATP content in the hypothalamus neurons, which regulate food intake of the body [132]. It is also used as an antidiabetic [133] and for the prevention of aspirin-induced gastric impairment [134].
6.	*Hypoxis hemerocallidea* Fisch., C.A.Mey. & Avé-Lall.	Hypoxoside (found in corms) [135,136]	Antitumor properties [137]
7.	*Pelargonium sidoides* DC.	Epicatechin [138].	Can inhibit mucin production in sputum and thus exhibit bronchodilator activity [139].
8.	*Siphonochilus aethiopicus* (Schweinf.) B.L.Burtt	Siphonochilone (from the roots and rhizomes) [140]. 1,8-cineole, cis alloocimene, (E)-β-ocimene, sabinene, terpinen-4-ol, kessane, and β-pinene [140].	Siphonochilone has potential in the treatment of asthma and allergic reactions [16]. Eucalyptol (or 1,8-cineole) has anti-inflammatory, muscle relaxant, analgesic, and antispasmodic activities [141]
9.	*Sclerocarya birrea* (A.Rich.) Hochst. subsp. caffra (Sond.) Kokwaro LC	Polyphenols, flavonoids, and condensed tannins	Can prevent chronic and degenerative disorders [142]
10.	*Warburgia salutaris (G.Bertol.) Chiov.*	drimane and colorotane sesquiterpenoids are the major constituents [143]. Warburganal [144], polygodial [145], salutarisolide [146], muzigadial [147], ugandensidial, isopolygodial [148], mukaadial [149] and mannitol [66]	Sesquiterpenoids have insect anti-feedant, anti-microbial, anti-cancer, molluscidal and anti-fungal activities [150]

**Figure 4:**
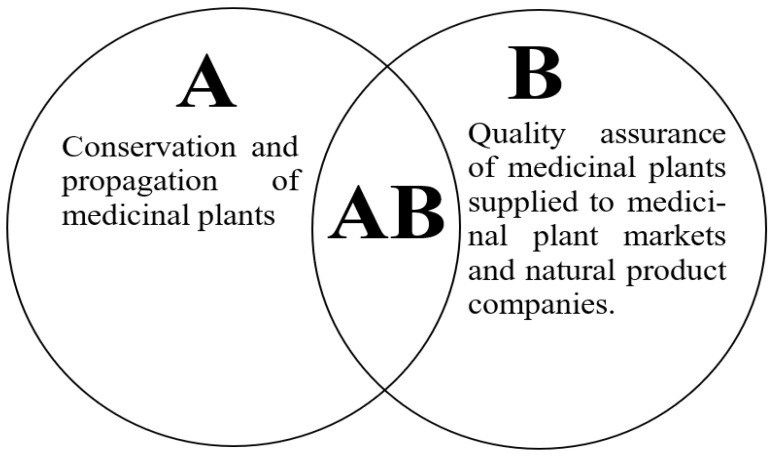
Conservation, propagation, and quality assurance as determining factors of the functionality of the traditional medicine system.

As illustrated in Figure 4, the conservation and propagation of medicinal plants (A) and quality assurance (B) of plant products are interwoven, determining factors regarding the functionality of traditional medicine systems (AB) (Figure 4). Major questions to be addressed are ‘Does the conservation of medicinal plants through propagation offer quality plant materials?’ and ‘which propagation method(s) is/are most suitable for each medicinal plant family?’. A decrease in the levels of major bioactive ingredients of plants raised in vitro equates to reduced efficacy of the plant material, while an increase in the major bioactive ingredient equates to increased efficacy. However, this increase may lead to health concerns, including cases of severe side effects or overdose. Hence, it is important to put control measures in place to ensure that standardized and quality medicinal plant materials are offered for sale in local markets and to natural product companies. To achieve this, the traditional medicine system needs to be restructured to implement the following:

1. Control the supply of plant materials to medicinal plant marketers.
2. Educate sellers about the dangers involved in the sale of sister species in place of the well-known potent species.
3. Involve academic stakeholders in determining the levels of bioactive compounds in plants produced through different propagation methods and their suitability as traditional medicines.
4. Enlighten medicinal plant marketers about the role of seasons on the plant’s major bioactive compound(s) composition, and
5. Establish networks and or platforms in which all stakeholders are members.

## 3. Materials and Methods

A literature search was conducted using electronic databases such as Scopus, Web of Science, Google Books, and Google Scholar. Other sources scrutinized for relevant information included student theses from South African Universities, published manuals and South African online news sources.

## 4. Conclusions and Recommendations

South African medicinal plants with a high potency often receive considerable attention from the international community, which, among other factors, leads to the conservation and propagation of medicinal plants. In the process of conservation and propagation, different stakeholders, including the academic community, are involved. The role of the academic community in the propagation and sustainability of South African medicinal plants cannot be over-emphasized. The roles of the academic stakeholders should further involve the development of new products from propagated plants. This can be achieved by a proper restructuring of the academic curriculum of plant biology/plant sciences at both undergraduate and postgraduate levels. An effective curriculum should include food processing and product development modules and some entrepreneurship modules. These steps will help the academic community to commercialize scientific results with ease and thus prevent valuable innovations from being lost to international companies or foreign countries. 

The economic growth of Africa can be achieved by a well-planned and efficient use of Africa’s natural resources, which includes the use of highly potent medicinal plants. This will go a long way to sustain rural households involved in the sale of medicinal plants. Hence, the economic development of the individuals in the lower economic pyramid could be achieved by prompting the use of natural resources within their environment. Insight into the way a community utilizes its natural resources, and factors influencing the degree of their use is essential for the development and implementation of policies for its sustainability and availability. This is crucial for the functionality of the African traditional medicinal system. 

## Data Availability

Data sharing does not apply to this article as no new data were created or analyzed in this study.
